# Under the Skin of a Lion: Unique Evidence of Upper Paleolithic Exploitation and Use of Cave Lion (*Panthera spelaea*) from the Lower Gallery of La Garma (Spain)

**DOI:** 10.1371/journal.pone.0163591

**Published:** 2016-10-26

**Authors:** Marián Cueto, Edgard Camarós, Pedro Castaños, Roberto Ontañón, Pablo Arias

**Affiliations:** 1 Instituto Internacional de Investigaciones Prehistóricas de Cantabria, Universidad de Cantabria, Santander, Spain; 2 Institut Català de Paleoecologia Humana i Evolució Social, Universitat Rovira i Virgili, Tarragona, Spain; 3 Sociedad de Ciencias Aranzadi, San Sebastián, Spain; 4 Museo de Prehistoria y Arqueología de Cantabria, Santander, Spain; Université de Poitiers, FRANCE

## Abstract

Pleistocene skinning and exploitation of carnivore furs have been previously inferred from archaeological evidence. Nevertheless, the evidence of skinning and fur processing tends to be weak and the interpretations are not strongly sustained by the archaeological record. In the present paper, we analyze unique evidence of patterned anthropic modification and skeletal representation of fossil remains of cave lion (*Panthera spelaea*) from the Lower Gallery of La Garma (Cantabria, Spain). This site is one of the few that provides Pleistocene examples of lion exploitation by humans. Our archaeozoological study suggests that lion-specialized pelt exploitation and use might have been related to ritual activities during the Middle Magdalenian period (ca. 14800 cal BC). Moreover, the specimens also represent the southernmost European and the latest evidence of cave lion exploitation in Iberia. Therefore, the study seeks to provide alternative explanations for lion extinction in Eurasia and argues for a role of hunting as a factor to take into account.

## Introduction

Cave lion (*Panthera spelaea* Goldfuss, 1810) fossils are present in the European Pleistocene archaeopaleontological record (e.g., [1,2] and references therein), although no strong evidence indicates a role for hominins in their accumulation or identifies anthropogenic modifications of the bones. Nevertheless, these carnivores developed an alternate use of caves with humans [3] and were responsible for some of the animal carcasses accumulated in cavities, mixed with anthropic assemblages [4,5], and for the destruction of their spatial connections [6]. Therefore, the interaction between hominins and lions and other large felines during the Pleistocene is a complex issue, and especially during the Upper Paleolithic, when they played a theoretically important role as attacking animals (e.g., [7,8]) or as symbolic animals in cultural traditions (e.g., [9–13]). In addition, some evidence supports the idea that these animals were also hunted by Paleolithic human groups (e.g., [14]).

The first evidence of direct fossil lion exploitation dates to the Middle Pleistocene, related to a *P*. *leo fossilis* sporadic hunting event [15]. More remarkable evidence for lion hunting and consumption does not emerge again from Europe until the Middle Paleolithic (e.g., [14]), although the evidence is still sparse, as previously mentioned. On the contrary, hunting and exploitation of other species of small and large carnivores (including ursids, felines, and canids) is widely documented (e.g., [16–21], see volume [22]).

During the Upper Paleolithic, general carnivore hunting increased [23], so small to large carnivores bones from that period display cut-marks and other anthropogenic modifications that can be related to different activities (bone breakage, cooking, knapping, etc.) and a much more diversified exploitation of usable resources (skin, tendons, meat, marrow, bone, teeth, etc.) (e.g., [18,19,24,25]). However, cave lion hunting is not commonly identified, except in the Swabian Jura (Germany) (e.g., [14,26,27]). Therefore, although cave lions are well known from a paleobiological point of view (e.g., [2] and references therein, [28–30]), their role in hominin-carnivore interaction processes is not yet clearly understood.

The site of the Lower Gallery of La Garma (*LG*) (Cantabria Spain) contains outstanding archaeological evidence due to the preservation of the original Paleolithic floors with no post-depositional modification due to sedimentation processes or spatial distribution. Our research analyzes key cave lion fossils from among the feline fossils from *LG*, with a representation of the skeletal parts, spatial distribution of the remains, and location of the anthropogenic modifications that allows us to infer unique evidence of human exploitation of *P*. *spelaea* in an archaeological context that appears to be related to Middle Magdalenian ritual. The relevance of this study is its contribution of new knowledge of direct interaction between humans and cave lions during the Upper Paleolithic.

From this perspective, our aim is to provide new data on hominin-carnivore interaction during the European Late Pleistocene, and to contribute to the debate on cave lion extinction (see [31]) and the hardly known unrevealed exploitation and use of cave lions by human groups.

### The Lower Gallery of La Garma

**Fig 1:**
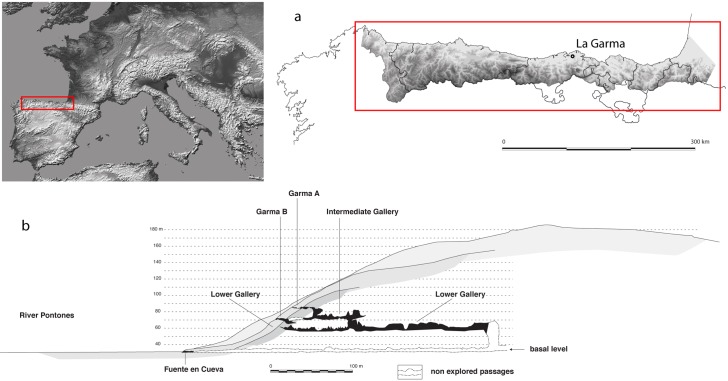
Map of the location of La Garma (a) and the N-S cross-section of the La Garma hill, with the karstic system depicted in black (b).

**Fig 2:**
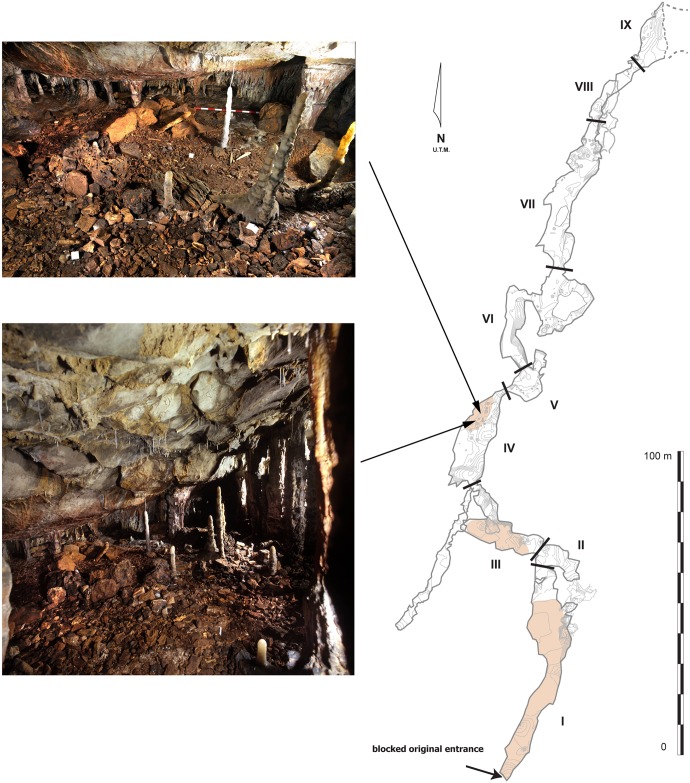
Plan of the Lower Gallery of La Garma, and images of the anthropic Paleolithic structures preserved in Zone IV.

The *LG* (Cantabria, Spain) (Fig 1a), discovered in 1995, is located in one of the four main levels of a karstic system; three of them have archaeological evidence and the fourth is a basal level (Fig 1b) [32]. Nevertheless, three other smaller galleries compose the system. The *LG* is a 300 m North-South rectilinear gallery. Its original entrance was blocked during the Late Pleistocene. Today, the entrance is located in the uppermost level, La Garma A, shown in Fig 1b) and because of this blockage, no sedimentary process has ever affected the last Upper Paleolithic human occupation so that outstanding evidence of intact prehistoric and original floors is preserved [33] (Fig 2). The *LG* has been divided in nine zones; Zones I, III, and IV are the ones with the highest concentrations of Paleolithic archaeological material (for further information about each zone see [34]).

**Fig 3:**
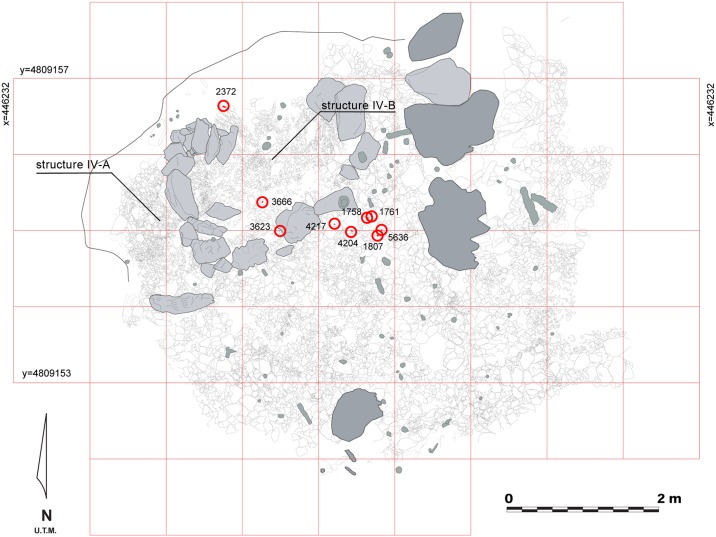
Plan of the anthropic structure from Zone IV in the Lower Gallery of La Garma, with the locations of the cave lion phalanxes circled in red.

This study examines cave lion remains located in Zone IV, where three anthropic stone structures, inferred to be short-term occupied Paleolithic huts, are located [34–36] (Figs 2 and 3; S1 Video). These structures are placed 130 m from the original entrance. Zone IV also contains evidence of previous human visits to the cave, such as rock art dated to the Gravettian period [37]. The archaeological complex was included as a World Heritage site by the UNESCO in 2008 [38].

Zone IV has been ascribed to the Middle Magdalenian and inferred to have a non-domestic ritual context [35]; it has been radiocarbon dated to 14300–14000 cal BP. [34]. It represents a single occupation, according the results of an archaeological test pit in the zone [34], and can be defined as an unmodified Paleolithic occupation floor (see discussion in [36]).

Over 4,000 mammalian faunal remains have been studied, with identified taxa including *Equus caballus*, *Bos* sp., *Cervus elaphus*, *Rangifer tarandus*, *Ursus arctos*, *Ursus* sp., *P*. *spelaea*, *Vulpes vulpes*, *Crocuta crocuta*, and Leporidae [34]. The presence of taphonomic modifications on the bone surfaces, such as cut-marks, bone breakage, and burned bones, in addition to the presence of non-carnivore marks, indicates the anthropic origin of the assemblage.

## Materials and Methods

### Paleontological Ethics Statements

All necessary permits were obtained for the present study by PA as director of the archaeological site, and complied with all relevant regulations. The permits were granted by Consejería de Educación, Cultura y Deporte of Government of Cantabria.

Specific site conservation standards required the analysis of the fossils *in situ*, with no permission granted to remove them from the site and with the stipulation that they be replaced in the same exact position after the analysis (S1 Fig).

The individual in this manuscript has given written informed consent (as outlined in PLOS consent form) to publish these case details.

### Materials

**Fig 4:**
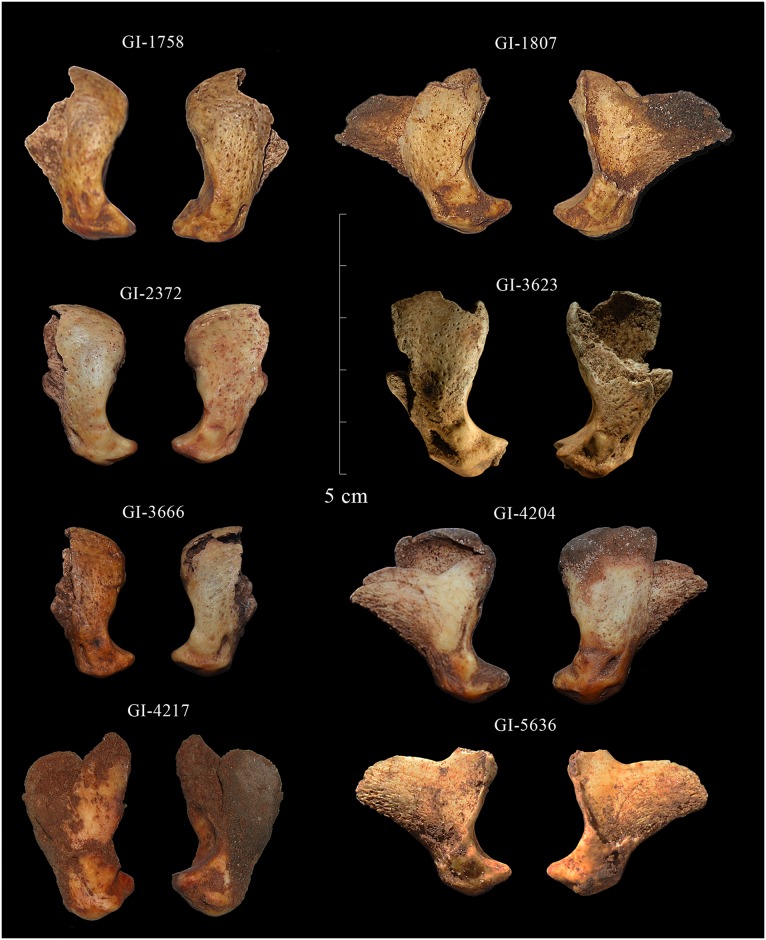
Cave lion distal phalanxes from the Lower Gallery of La Garma. Note that only eight of nine specimens are depicted in the figure.

**Fig 5:**
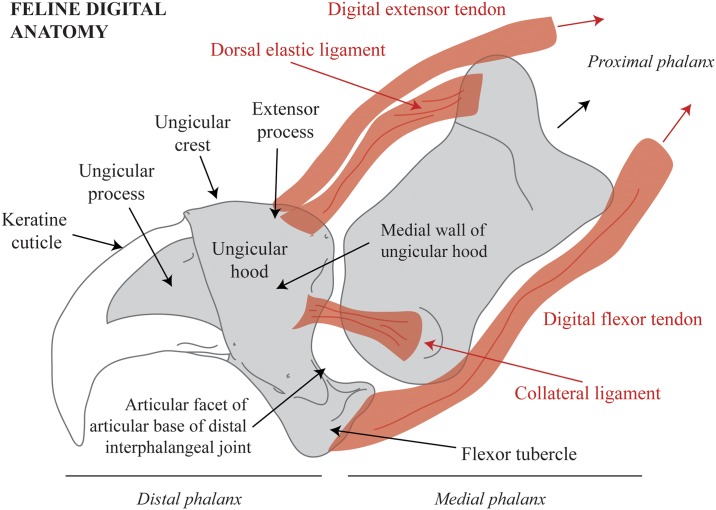
Lateral view of a schematized distal and medial feline phalanx in anatomical connection. Tendons and ligaments are depicted in red.

A total of nine faunal remains belonging to *P*. *spelaea* (Goldfuss, 1810) have been identified in Zone IV from the *LG* (Fig 4). The anatomy, taxonomy, and taphonomic modifications have been studied following the standard archaeozoological methods (see [39–41]), and the nomenclature used has been taken from Homberger et al. [42] (Fig 5). The morphology of cut-marks (curved/V-shaped) was analyzed.

All specimens were treated at both macroscopic and microscopic levels at the site (except for a single specimen that remained attached to the floor due to concretion). Specific site conservation standards required the analysis of the fossils *in situ*, with no permission granted to remove them from the site and with the stipulation that they be replaced in the same exact position after the analysis (S1 Fig). A better taphonomical characterization of the bone surfaces, not possible due to the working conditions in the cave (described in [34,36]), was achieved using the non-destructive technique of modeling and casting bones for the stereomicroscopic analysis described by Camarós et al. [43]. Previous research has proven the potential for producing high-resolution transparent casts for the observation of taphonomic modifications on bone surfaces [23]. Casts were analyzed using a stereomicroscope with transmitted light (Zeiss Stemi 2000C).

The specific cave conditions, such as temperature (≈13,5 C°) and humidity (≈95%), necessitated modifications of the specific timings used in the molding protocol. The high-resolution silicone set after 19 minutes and the low-resolution silicone was then placed over and separated from the bone surface after an additional 9 (S2 Fig).

A single specimen [GI-3623 (Fig 4)] was directly radiocarbon-dated at the Oxford Radiocarbon Dating Laboratory using two bone samples. Calibrations are referred to the IntCal13 curve [44] and modelled in OxCal v.4.2. [45].

The ^14^C dates obtained are: 13830±55 BP (OxA-18698) and 13835±60 BP (OxA-18699), providing the pooled mean date of 13832±41 BP. They are statistically identical [15017–14563 cal BC (95.4%); 14921–14673 cal BC (68.2%)]. This Radiocarbon dates are consistent with the other dates from this context [34].

## Results

The nine *P*. *spelaea* remains analyzed in this study (Fig 4) represent 0.23% of the total animal bone assemblage (NR 3,928). They are the only lion bones identified in Zone IV of the *LG*. All are distal phalanxes from unidentified anterior or posterior extremities and unknown side. As discussed later, all these remains belong to a unique adult individual of unidentified sex. The observation of the articular facet of the distal phalanxes suggests that the specimen from *LG* belongs to the small morphotype of small sized lions common in Cantabrian Spain [46]. A generalized evolutionary size reduction has been noted during the Pleistocene by Marciszak and colleagues) [2]. One sample has been radiocarbon-dated twice, chronologically ascribing it to the Middle Magdalenian.

**Fig 6:**
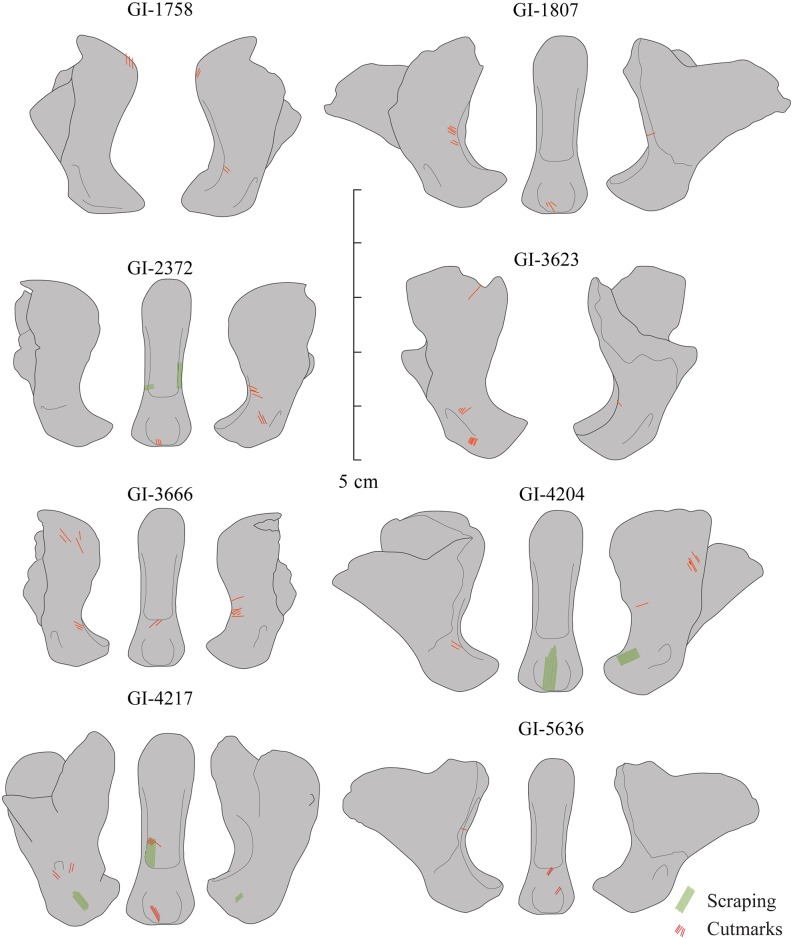
Cave lion phalanx drawing showing the observed anthropic modifications on the bone surface.

The taphonomic study reveals that all nine bones are relatively well preserved; most of them even have maintained the presence of the unguicular process, the weakest part of the bone, although none of them have preserved horn caps (keratin cuticle). Eight out of nine distal phalanxes display observable cut-marks, most of them in the articular facet (Fig 6). No other kinds of taphonomic modifications have been recorded, due to post-depositional carbon calcite concretion formed on some of them. Characterization of the anthropogenic modification pattern required special emphasis on the taphonomic individualization of each specimen.

### Phalanx specimen GI-1758

**Fig 7:**
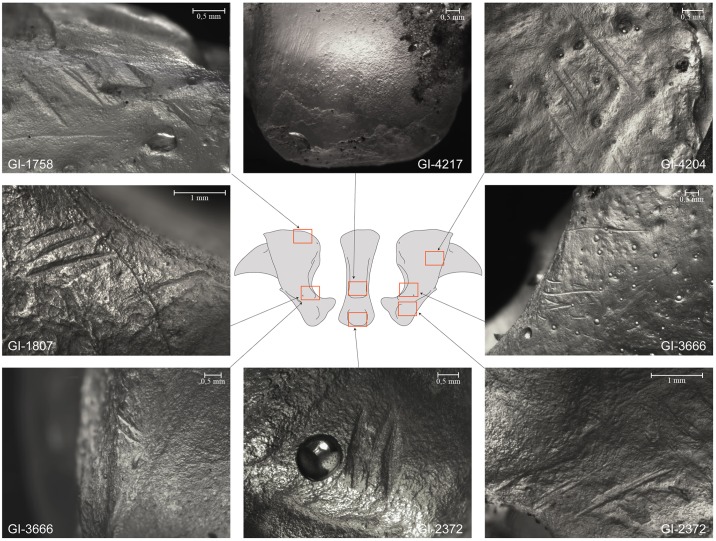
Anthropic modifications recorded in some of the specimens as observed on the transparent silicone casts.

This specimen does not preserve the unguicular hood (Fig 4) and displays small oblique cut marks on both sides of the proximal zone of the unguicular hood, just at the attachment of the extensor process (Fig 7), as well as on the right side of the articular facet of the articular base of the distal interphalangeal joint (Fig 6).

### Phalanx specimen GI-1761

This specimen, although anatomically and specifically identifiable, displays concretion and is attached to the floor stones. Therefore, analysis has been impossible, and any anthropic modification that may be present on the bone surface cannot be discerned.

### Phalanx specimen GI-1807

This phalanx is considerably complete and only the right side of the unguicular hood wall is not preserved (Fig 4). It displays two grouped, small, parallel cut-marks on the left border of the articular facet and oblique ones on the exact opposite side (Fig 7), as well as on the central distal part of the flexor tubercle (Fig 6).

### Phalanx specimen GI-2372

Phalanx GI-2372 does not preserve the totality of the unguicular process (Fig 4). Modifications include both cutting and scraping traces (Fig 6). Cut-marks are defined by small grouped oblique cuts located on the border of the articular facet and the flexor tubercle, on the right lateral surface of the wall, and on the central part seen from a posterior view (Fig 7). Scraped zones are restricted to the articular facet of the articular base of distal interphalangeal joint.

### Phalanx specimen GI-3623

This specimen does not preserve the totality of the unguicular process and the right side of the unguicular hood wall (Fig 3). Cut-marks were identified on the proximal area of the wall of the unguicular hood, near the extensor process, and on both sides of the tuberosity on the right lateral side of the flexor tubercle. Cutting marks also occurred on the opposite side, near the border of the articular facet (Fig 6).

This phalanx has been dated by ^14^C, and it is no longer preserved (see Material and Methods section).

### Phalanx specimen GI-3666

This phalanx does not preserve the totality of the unguicular process, and the unguicular crest and extensor process are badly preserved (Fig 4). The specimen has cut-marks on the proximal left zone of the unguicular hood. Cutting traces are also evident on the right and left borders of the articular facet (Fig 7), and on the border of the central area of the flexor tubercle, seen from a posterior view. All anthropic modifications are distributed in groups of parallel small and oblique cut-marks (Fig 6).

### Phalanx specimen GI-4204

This phalanx is considerably complete and only the left side of the unguicular hood wall is not preserved. The specimen displays cut-marks, as well as scraped zones (Fig 6). Cutting traces are two oblique and small grouped lines located on the right medial wall of the unguicular hood (Fig 7) and on the left side of the flexor tubercle zone. Intensive scraping is observable on the central part of the tubercle base, resulting in a considerable loss of bony material, and on the right side of the border of the articular facet of the articular base of the distal and medial phalanxes.

### Phalanx specimen GI-4217

This specimen does not preserve the totality of the unguicular process and some parts of the medial wall of the unguicular hood (Fig 4). Nonetheless, cut-marks and scrapings have been recorded (Fig 6). Cutting traces are defined by small grouped parallel oblique cuts located on the left wall of the articular base, near the tubercle, and on the left border of the articular surface. Cut-marks are also observed on the flexor tubercle. Scraped zones are found on both sides of the lateral area of the tubercle and on the articular facet (Fig 7).

### Phalanx specimen GI-5632

This phalanx does not preserve the unguicular hood on both sides (Fig 4), and the anthropogenic modifications are cut-marks restricted to the left border of the articular facet and to the central area of the flexor tubercle, seen from a posterior view (Fig 6). Traces can be described as grouped parallel oblique cuts.

## Discussion

Zone IV of the Lower Gallery of the site of La Garma is a unique archaeological assemblage due to its outstanding preservation and the lack of sedimentary processes affecting the material [34–36]. Therefore, its archaeological inferences, and especially those related to its spatial distribution and the presence of cultural objects (e.g., [32,34]), represent an important advance in the conception of human behavior during the Upper Paleolithic.

Faunal remains from *LG* are the most abundant category of materials, providing high potential for approaching behavioral issues when an archaeozoological analyses are conducted [34]. This has been proved by studying cave lion fossils recovered at the site, allowing us to contribute to the debate on Upper Paleolithic subsistence and faunal exploitation strategies, as well as in those linked with behavioral and ritualistic aspects of human cognitive evolution.

The cave lion phalanxes represent rare and unique evidence of both taxonomical presence and carnivore exploitation during the Upper Paleolithic. Among the analyzed faunal assemblage, the only lion anatomical elements identified are distal phalanxes, most of them with anthropic modifications. Few cave lion fossils have been recovered from the Upper Paleolithic sites in Cantabrian Spain (e.g., [31,46–48]), and they do not display human modification. In Western Europe there are also cave lion fossils (e.g., [49] and references therein, [50,51]) but only a few has human modifications [52]. Those remains with evidence of anthropic taphonomic damage indicate exceptional lion hunting events and exploitation related to use of the skin, tendons, and teeth as raw materials [14,27]. In this sense, cave lion hunting is not highly associated with subsistence strategies, again suggesting sporadic exploitation. Nevertheless, the presence of cave lion remains (including those with anthropogenic modifications) is not sufficient to infer clear exploitation patterns and behavioral implications, and this is precisely the contribution of the specimens studied by us toward the understanding of Paleolithic exploitation of large Pleistocene felines.

**Fig 8:**
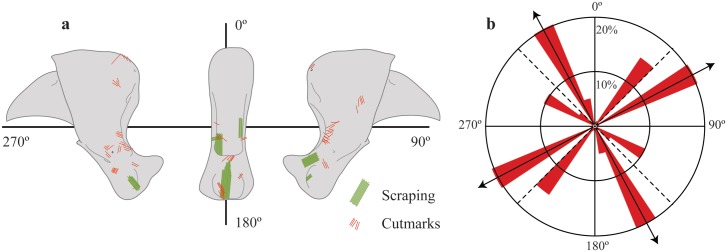
Summary of the anthropic modifications among all specimens analyzed (a) and a rose diagram showing the orientation of cut-marks on the phalanxes (b).

Our results show how human modifications of the cave lion phalanxes from the *LG* display an evident exploitation pattern. Overall, these modifications, produced with lithic tools, can be grouped into specific areas of the claw anatomical structure, thereby revealing cutting preferences and well-defined actions. As Fig 8a shows, most of the cut-marks and scraping traces are located on the lateral and posterior facets of the flexor tubercle of the articular base. Nevertheless, a few cut-marks are found on the medial and proximal wall of the unguicular hood. All cut-marks reveal a cutting action at an angle from the proximal to distal areas, in a repeated action, demonstrating an in-depth knowledge of animal processing and anatomy (as seen in the rise in the cut-mark orientation in Fig 8b). All recorded modifications are interpreted as the result of transectional cuts at the distal interphalangeal joint for the disarticulation of both phalanxes. In sum, we can infer a reiterative, well-patterned, and experienced action of cutting the dorsal elastic and collateral ligaments and the deep digital flexor tendon. An interesting note is that these marks are present in same zones associated with the modern veterinary operation of declawing (onychectomy surgery) of domestic felines [53]. Moreover, this is also the technique used by modern hunters when skinning their prey when the aim is to keep the claws attached to the fur.

The fact that the number of phalanxes recovered does not match the estimated number of elements for a single individual (a total of 18 expected distal phalanxes) may be explained by a loss of elements before the introduction of the pelt into the site. Another explanation could be that we have not recovered all the phalanxes, which may be hidden in the floor below the rocks at *LG*, although this is not the most likely explanation. A further possibility is that only the anterior claws were part of the pelt (a total of 10 expected distal phalanxes) and therefore only one is absent. This is the most plausible explanation because in an extended skin, the exposed part of the hind legs is the back of the paw, so the claws are not visible if esthetic criteria are followed.

Distal phalanxes are never measured for biometrical analysis due to their high variability among the same individuals. For this reason, no comparative data are available to determine if our specimens are consistent with the presence of a single lion at the site. Therefore, our interpretations regarding the presence of a unique animal are based on the number of phalanxes, the spatial distribution, the absence of more lion remains, and the archaeological context.

The presence of carnivores in Pleistocene and Holocene sites is commonly associated with fur use (e.g., [54–65]). The inferred exploitation patterns can be linked with large and small carnivore pelt exploitation (e.g., [15,17,19,25,66,67]), according to historic, ethnographic, and actualistic observations for fur procurement resulting in usable carnivore skins [68–70], which are also applicable to the *LG* cave lion remains.

Importantly, confirming skinning activities is a problematic issue, according to several authors [66,70,71] due to a large number of factors, including processes affecting the original spatial distribution and excavation procedures. In the *LG*, these factors do not affect our interpretations and therefore they are also reinforced by the spatial distribution and a non-biased skeletal-element representation. In this sense, the presentation of the unguicular hood, which is a very weak zone, confirms that the observed spatial distribution has not been affected by anthropic modifications occurring after deposition. In turn, this reveals the use of the fur in the last occupation of the cavity. Furthermore, we associate our cave lion remains with existing theoretical models for fur exploitation and the introduction of pelts into campsites, which would result in the overrepresentation of skeleton extremities and the lack of other skeletal parts (see [70,72–74]).

Furthermore, the spatial distribution of the phalanxes (Fig 3) reveals a coherent spatial connection among the remains that is consistent with the presence of at least one lion pelt (with paws attached) that had been transported to the site after processing following a patterned and specialized procedure, with no relation to subsistence strategies or ornament fabrication. The fact that some of the phalanxes are located in the interior of the hut, while others are in the outside area, could help in understanding the use of the pelt. If we assume that a unique cave lion skin is represented, the fur could also have had a function related to covering the floor or the structure due to its spatial distribution. Other functionalities beyond clothing, such as hut structural elements like covertures, have been interpreted previously for other Upper Paleolithic sites, based on ethnographic analogies [75–81].

The hypothesis of the presence of a single lion would suggest a sporadic, isolated, and rare event of large carnivore hunting, as indicated (although other scenarios cannot be excluded, like scavenging) in ancient chronologies [15]; however, the well-defined pattern inferred from the locations of the cut-marks and scraping traces permits us to infer an experienced procedure and a high knowledge of animal anatomy. Furthermore, the presence of the remains of other carnivores, such as bears (*Ursus arctos*), at the site that also show anthropic modifications, reveal the successful hunting of dangerous carnivores, as has also been observed at other Magdalenian sites (e.g., [52,67,82,83]).

The potential linkage of the presence of a cave lion skin in such an archaeological context with ritual activities [35] allows us to infer a probable important role of the cave lion during the Magdalenian period among human groups. Previous research has also linked large felines and symbolic issues, such as the one conducted with the Aurignacian ivory figurines from the Swabian Jura (Germany) [9,13,84]. Upper Paleolithic graphic expression also clearly places lions in a prominent hierarchic position in the early humans’ symbolic world, giving this animal an important role in human culture as a motif (e.g., [10,85,86]). Modern ethnographic sources also connect lions with symbolic beliefs, especially through manhood rituals and prestige hunting activities with inherent risks, as conducted, for example, by the Maasai people [87]. Other human groups did the same with other carnivores, such as bear hunting for obtaining furs, in different prehistoric and historic periods (e.g., [88,89] and references therein). This mythification of dangerous prey could explain the evidence in the Upper Paleolithic cave of lion hunting and pelt exploitation in Zone IV of *LG*, as it could have been motivated by ideological considerations that justify its presence in a context interpreted as the practice of ritual activities [35,36].

Cave lion remains from the *LG* also provide interesting information related to paleoecological issues and the role of humans as a factor of pressure in ecological niches. *P*. *spelaea* was widely distributed in Eurasia [90] during the Late Pleistocene, and it was also present in the Iberian Peninsula, basically restricted to its northern area [46]. Our remains are interesting in this biogeographical context, as they indicate how cave lions persisted in northern Spain following the Last Glacial Maximum, as other directly dated remains from Urtiaga Cave show (17032 cal BP (95.4%)(OxA-10121) in [31]). Nevertheless, they also relate to general lion extinction in Eurasia, which occurred ca. 14500–14000 cal BP [31], although new and necessary direct radiocarbon dating of *P*. *spelaea* fossils could reduce its persistence up to chronologies near the Holocene.

One point that can be made is that the fossils studied for the present paper do not solely represent a unique archaeological proof of carnivore exploitation and use during the Paleolithic; rather, they represent the latest evidence of cave lion exploitation during the Upper Paleolithic. In addition, they represent the southernmost European presence of *P*. *spelaea* during the Late Pleistocene and one of the westernmost indications (see map provided by [31]) at a latitude and longitude of 43°25′50″N 3°39′57″W. Although these remains are not sufficient to allow conclusive statements to be made, they sum the data to highlight the role of human activity in the extinction of carnivores, as has been suggested for other geographical areas, in addition to other factors such as climate change, prey numbers, or species replacement (e.g., [23,30], also summarized in [31]).

Modern case studies have demonstrated that a decrease in extant African lions can be related to direct human intervention through rapid habitat destruction, depletion of resources, and over-hunting [91,92]. In this sense, we associate this well-defined lion skinning exploitation pattern from *LG* with a previously practiced activity reached through intense hunting during the Upper Paleolithic. A tentative proposal might be to link this knowledge to an important role in human culture resulting in a key factor that should be taken into account to understand cave lion extinction.

In sum, the *LG* appears to be an important site that can contribute to the study of complex hominin-carnivore interactions during the Upper Paleolithic. This site allows researchers to approach behavioral implications in a context of ritualistic activities or to examine the role of humans in large carnivore extinctions, due to its outstanding and well-preserved archaeological assemblage.

## Conclusions

Zone IV in the *LG* (Cantabrian Spain) is an archaeological ritual context that preserves outstanding Magdalenian evidence due to its non-modified anthropogenic assemblage [34–36], and it has a high potential to approach behavioral issues.

It is by no means clear that our results point towards a unique and unequivocal archaeological evidence of cave lion exploitation event during the Upper Paleolithic, with the aim of skinning the feline and use its fur in a ritual context. The representation of skeletal parts, in addition to the spatial distribution of the faunal remains and the nature and location of the anthropogenic modifications on bones with no archaeological parallels, reinforces this interpretation. This explanation also accounts for previous theoretical patterns and infer carnivore fur use and skinning practices; therefore, they are the only recognizable and distinct evidence of such use during the Pleistocene.

In conclusion, we suggest that this outstanding evidence of specialized and patterned skinning exploitation of cave lions in the Upper Paleolithic, as inferred in *LG*, can be viewed as a complex hominin-carnivore interaction scenario. Its association with ritual activities provides key evidence for approaching behavioral issues in relation to cultural traditions and speculative alternative explanations to cave lion extinction during the Late Pleistocene, assuming a role for human hunting as a determining factor, among others. Further research will be needed to test this hypothesis, for answer to the questions addressed in order to contribute to the debate with new data, although the problem is a complex one.

## Supporting Information

S1 Fig Cave lion distal phalanxes from the Lower Gallery of La Garma in their original position. (TIF)

S2 Fig The modeling process adapted to the interior specific conditions of Lower Gallery of La Garma. A. The modeling process at Lower Gallery; B. Testing the method with a bone from archaeological site; C. Cleaning the bone surface of the distal phalanx with ethanol; D. Applying the high-resolution silicone directly to the bone surface; E. Covering the negative impression with low-resolution silicone; F. Transparent casts made by epoxy resin, prepared to analyze by stereomicroscope with transmitted light.
(TIF)

S1 Video Structures IV-A and IV-B from the Lower Gallery of La Garma. (M4V)
