# Supplementary figures and images for: Under the Skin of a Lion: Unique Evidence of Upper Paleolithic Exploitation and Use of Cave Lion (Panthera spelaea) from the Lower Gallery of La Garma (Spain)
Source: PLoS One. 2016 Oct 26;11(10):e0163591. doi: 10.1371/journal.pone.0163591 (PMC5082676; doi:10.1371/journal.pone.0163591)

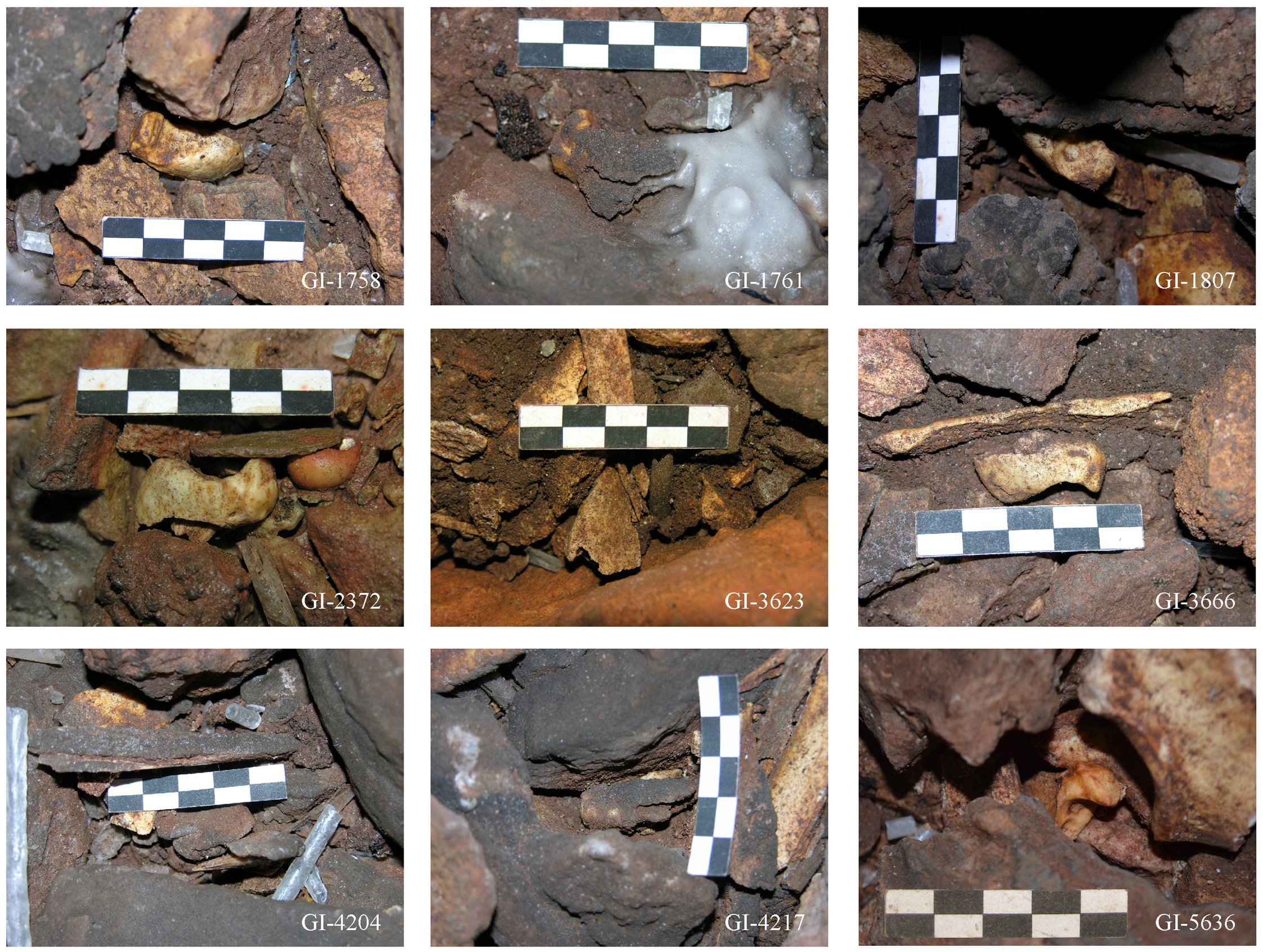

Supplement: S1 Fig — (TIF) [file pone.0163591.s001.tif]

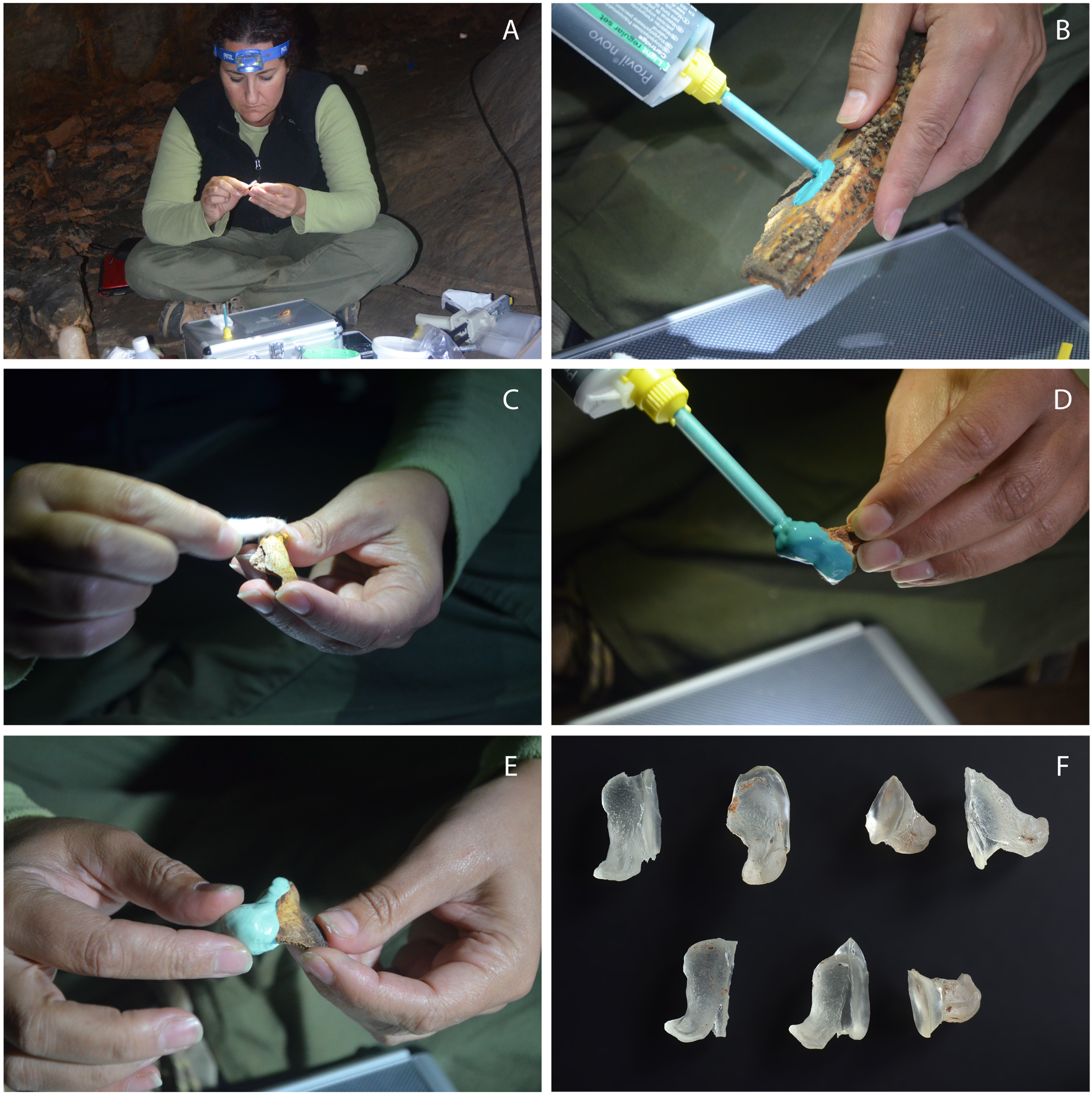

Supplement: S2 Fig — A. The modeling process at Lower Gallery; B. Testing the method with a bone from archaeological site; C. Cleaning the bone surface of the distal phalanx with ethanol; D. Applying the high-resolution silicone directly to the bone surface; E. Covering the negative impression with low-resolution silicone; F. Transparent casts made by epoxy resin, prepared to analyze by stereomicroscope with transmitted light. (TIF) [file pone.0163591.s002.tif]
